# Supplementary material for: High seroprevalence of antibodies to Dengue, Chikungunya, and Zika viruses in Dire Dawa, Ethiopia: A cross-sectional survey in 2024
Source: PLoS Negl Trop Dis. 2025 Jul 28;19(7):e0013357. doi: 10.1371/journal.pntd.0013357 (PMC12352872; doi:10.1371/journal.pntd.0013357)
Supplement: S1 Text — Fig A. Age-specific seroprevalence by arbovirus and for IgG and IgM Page 2. Fig B. IgG responses for ZIKV and DENV by test status Page 3. Table A. Crude serological results from Addis Ababa by age group, virus, and antibody type Page 4. Table B. Linear mixed models for IgG (random intercept for household) Page 5. Table C. Linear mixed models for IgM (random intercept for household) Page 6. Description A. Power Analysis. Sample size calculation methodology for Dire Dawa sero-survey Page 7. Description B. Statistical Modeling of Age-Seropositivity Relationships. GEEs with splines for IgG and IgM Page 8. Description C. Linear Mixed-Effects Modeling of Antibody Responses and Cross-Reactivity. Page 9. (DOCX) [file pntd.0013357.s001.docx]

**S1 Text**

### ****Table of Contents****

1. **Fig A in S1 Text.** Age-specific seroprevalence by arbovirus and for IgG and IgM Page 2
2. **Fig B in S1 Text.** IgG responses for ZIKV and DENV by test status Page 3
3. **Table A in S1 Text.** Crude serological results from Addis Ababa by age group, virus, and antibody type Page 4
4. **Table B in S1 Text.** Linear mixed models for IgG (random intercept for household) Page 5
5. **Table C in S1 Text.** Linear mixed models for IgM (random intercept for household) Page 6
6. **Description A in S1 Text. Power Analysis.** Sample size calculation methodology for Dire Dawa sero-survey Page 7
7. **Description B in S1 Text. Statistical Modeling of Age-Seropositivity Relationships.** GEEs with splines for IgG and IgM Page 8
8. **Description C in S1 Text. Linear Mixed-Effects Modeling of Antibody Responses and Cross-Reactivity.** Page 9

**Fig A in S1 Text:** Age specific seroprevalence by arbovirus and for IgG (top panel) and IgM (bottom panel). Bar size is the proportion who tested positive (“reactive”) by arbovirus. The corresponding whiskers are Wilson score confidence intervals. The main figure in the text has age groups in larger age bins.

Age specific seropositivity for dengue IgG was high across most age groups, beginning at around 25% in the 0 to 4 age group and reaching a high of approximately 90% in the 30 – 34 age group. Chikungunya IgG seropositivity was next highest, reaching a high of 75% in the 50 – 54 age group. As with both dengue and chikungunya, Zika IgG seropositivity was lowest in the 0 – 4 age group, increasing with age but leveling off around age 15 – 25. All age groups exhibited IgG seropositivity to all three viruses.

**** IgM to dengue virus and chikungunya virus was higher than for Zika virus, though all three were present in almost all age groups (with an exception of Zika IgM being absent in the 0 – 4, 20 – 24, 30 – 34, and 40 – 49 age groups). Dengue IgM seropositivity was dominant in age groups up until the 40 – 45 age group, when chikungunya IgM seropositivity became highest.

**Fig B in S1 Text:**

IgG responses for ZIKV and DENV by test status. Since all ZIKV IgG positives were also DENV IgG positive, there is no way to look at the IgG among ZIKV IgG positives but DENV IgG negative (panel A). Panel B shows, for all individuals who were ZIKV IgG negative, the differences in quantitative ZIKV IgG response among those who were DENG IgG positive (blue/green) and those who were DENG IgG negative (orange). Panel C shows, for those who were DENG IgG positive, the difference in quantitative DENG IgG values for those who were ZIKV IgG positive or negative. DENV IgG was much higher among individuals who tested positive for ZIKV IgG (panel C). ZIKV IgG is also higher among those who tested positive for DENV IgG (panel B), but the difference is small in comparison to association between

**
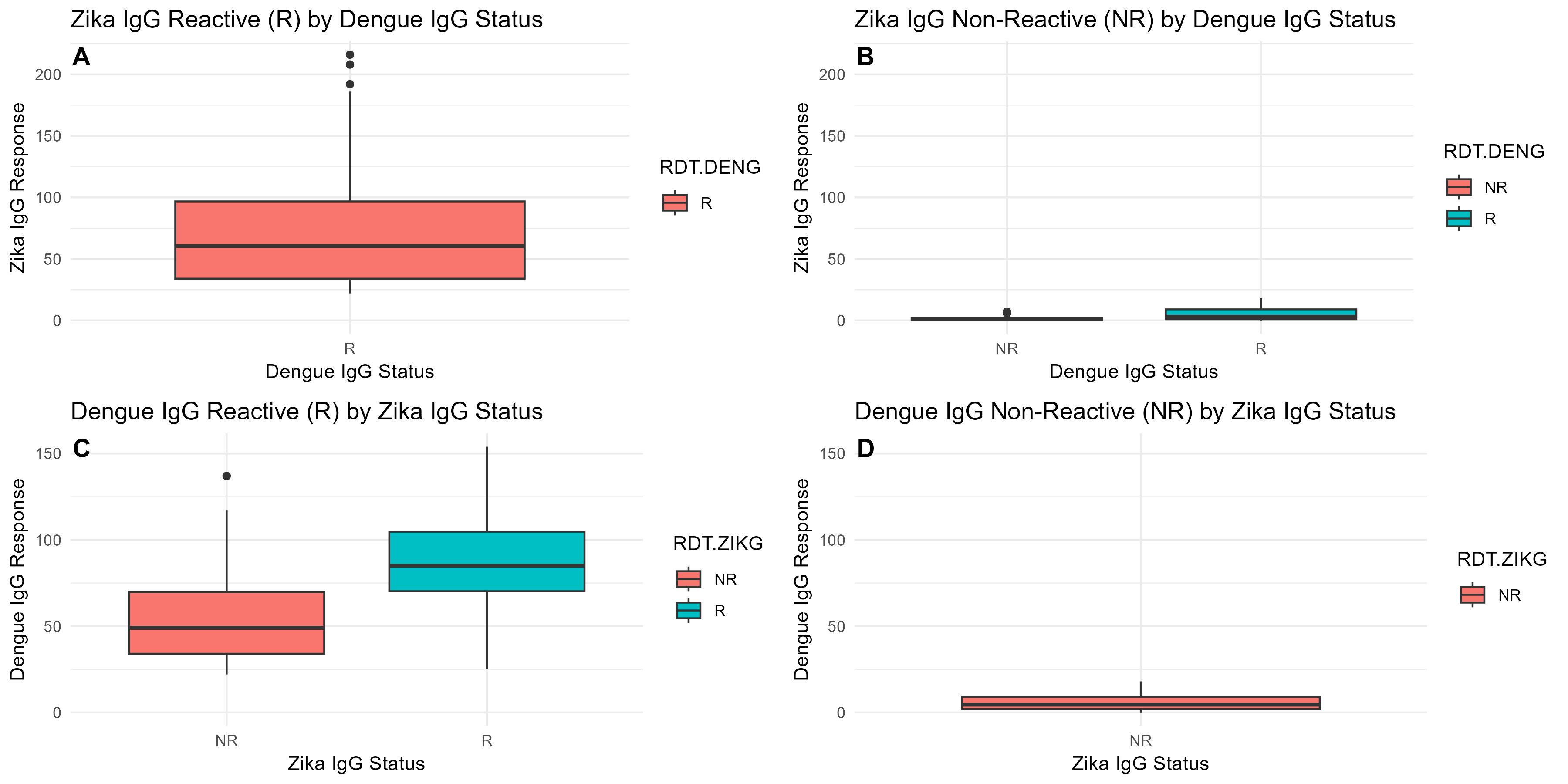
**

**Table A in S1 Text: Crude serological results from Addis Ababa by age group, virus, and antibody type (IgG or IgM).** Counts represent the number of individuals testing positive for IgG or IgM antibodies using the DPP ZCD rapid test. This survey was conducted as a type of comparison for the main survey in Dire Dawa (Table 1).

|  | **IgG** | | | **IgM** | | | **Total tested** |
| --- | --- | --- | --- | --- | --- | --- | --- |
| **Age Group** | **Dengue** | **Zika** | **Chikungunya** | **Dengue** | **Zika** | **Chikungunya** |  |
| 0-4 | 0 | 0 | 0 | 0 | 0 | 0 | 1 |
| 5-9 | 0 | 0 | 0 | 0 | 0 | 0 | 2 |
| 10-19 | 0 | 0 | 0 | 0 | 0 | 0 | 13 |
| 20-29 | 1 | 0 | 1 | 4 | 0 | 1 | 101 |
| 30-39 | 4 | 2 | 0 | 2 | 1 | 0 | 39 |
| 40-49 | 0 | 0 | 0 | 0 | 0 | 0 | 15 |
| 50-59 | 0 | 0 | 0 | 0 | 0 | 0 | 9 |
| 60-69 | 0 | 0 | 0 | 0 | 0 | 0 | 0 |
| 70+ | 0 | 0 | 0 | 0 | 0 | 0 | 0 |

**Table B in S1 Text: Linear mixed models (random intercept for household) for IgG.** The The “Model” column specifies which virus-specific IgG response was modeled as the outcome, while the “Covariate” column lists the IgG responses to other viruses included as predictors in that model.

| **Model** | **Covariate** | **Estimate** | **CI** | **Std.Error** | **p-value** |
| --- | --- | --- | --- | --- | --- |
| Dengue IgG | (Intercept) | 32.10 | (27.47, 36.73) | 2.36 | <0.0001 |
| Dengue IgG | Zika IgG | 0.52 | (0.45, 0.59) | 0.04 | <0.0001 |
| Dengue IgG | Chikungunya IgG | 0.14 | (0.09, 0.18) | 0.02 | <0.0001 |
| Zika IgG | (Intercept) | -5.61 | (-12.11, 0.89) | 3.31 | 0.0915 |
| Zika IgG | Dengue IgG | 0.72 | (0.62, 0.82) | 0.05 | <0.0001 |
| Zika IgG | Chikungunya IgG | -0.07 | (-0.13, -0.02) | 0.03 | 0.0087 |
| Chikungunya IgG | (Intercept) | 23.85 | (11.20, 36.50) | 6.46 | 0.0003 |
| Chikungunya IgG | Zika IgG | 0.70 | (0.47, 0.93) | 0.12 | <0.0001 |
| Chikungunya IgG | Dengue IgG | -0.27 | (-0.48, -0.07) | 0.10 | 0.0084 |

**Table C in S1 Text: Linear mixed models (random intercept for household) for IgM.** The The “Model” column indicates the antibody outcome (Dengue, Zika, or Chikungunya IgM), while the “Covariate” column lists the IgG and IgG responses to other viruses included as predictors in that model.

| **Model** | **Covariate** | **Estimate** | **CI** | **Std.Error** | **p-value** |
| --- | --- | --- | --- | --- | --- |
| Dengue IgM | (Intercept) | 7.99 | (6.52, 9.47) | 0.75 | <0.0001 |
| Dengue IgM | Zika IgM | 0.33 | (0.19, 0.47) | 0.07 | <0.0001 |
| Dengue IgM | Chikungunya IgM | 0.39 | (0.29, 0.49) | 0.05 | <0.0001 |
| Dengue IgM | ZIKa IgG | 0.06 | (0.04, 0.09) | 0.01 | <0.0001 |
| Dengue IgM | Chikungunya IgG | -0.06 | (-0.08, -0.03) | 0.01 | <0.0001 |
| Zika IgM | (Intercept) | 0.09 | (-1.39, 1.57) | 0.76 | 0.9050 |
| Zika IgM | Dengue IgM | 0.24 | (0.16, 0.32) | 0.04 | <0.0001 |
| Zika IgM | Chikungunya IgM | 0.18 | (0.10, 0.26) | 0.04 | <0.0001 |
| Zika IgM | Dengue IgG | 0.01 | (-0.01, 0.04) | 0.01 | 0.2430 |
| Zika IgM | Chikungunya IgG | -0.03 | (-0.05, -0.01) | 0.01 | 0.0013 |
| Chikungunya IgM | (Intercept) | 3.43 | (0.39, 6.47) | 1.55 | 0.0281 |
| Chikungunya IgM | Dengue IgM | 0.42 | (0.27, 0.58) | 0.08 | <0.0001 |
| Chikungunya IgM | Zika IgM | 0.41 | (0.20, 0.62) | 0.11 | 0.0002 |
| Chikungunya IgM | Dengue IgG | 0.06 | (0.01, 0.12) | 0.03 | 0.0178 |
| Chikungunya IgM | Zika IgG | -0.08 | (-0.12, -0.03) | 0.02 | 0.0013 |

**Description A in S1 Text:**

**Power analysis for Dire Dawa sero-survey**

We estimated the required sample size for a cross-sectional seroprevalence survey using the standard formula for a single population proportion, assuming a large or infinite population size:

n = (Z² × p × (1 − p)) / d²

Where:

n is the required sample size,

Z is the standard normal deviate corresponding to the desired confidence level (e.g., 1.96 for 95%),

p is the estimated population seroprevalence,

d is the desired margin of error (precision).

This formula assumes a large population and does not include a finite population correction. We selected conservative values for p and d to ensure adequate power to detect moderate to high seroprevalence levels, while accounting for design considerations such as clustering and non-response in later adjustments.

**Description B in S1 Text:**

**Statistical Modeling of Age-Seropositivity Relationships**

We used generalized estimating equations (GEEs) to estimate the age-specific probability of IgG and IgM seropositivity for dengue, chikungunya, and Zika viruses. GEEs account for clustering within households, where individuals may share similar exposure risks. For each outcome, we fit a logistic regression model with household ID as the clustering variable and modeled age as a continuous predictor using a single natural cubic spline with 3 degrees of freedom to allow for flexible, non-linear associations.

The model took the form:

logit(P(Yᵢⱼ = 1)) = β₀ + f(Ageᵢⱼ)

Where:

Yᵢⱼ is the binary serostatus (positive or negative) for individual i in household j,

Ageᵢⱼ is age in years,

f(Ageᵢⱼ) is the natural cubic spline transformation of age (df = 3),

An exchangeable correlation structure was used to model intra-household dependence.

Predicted probabilities were calculated by applying the logistic function to the linear predictor obtained from the model across a continuous age range. Standard errors were computed using the model matrix and the estimated variance-covariance matrix. Ninety-five percent confidence intervals for the predicted probabilities were obtained by transforming the linear predictor ±1.96 standard errors using the inverse logit function. These probabilities and confidence bands were visualized separately for each virus and antibody type. All analyses were conducted in R using the geepack and splines packages.

All statistical analyses were conducted in R using the *geepack* package for GEEs, *splines* for natural cubic spline terms, and *ggplot2* for visualization. Data handling and figure assembly used *dplyr* and *gridExtra*.

**Description C in S1 Text:**

**Linear Mixed-Effects Modeling of Antibody Responses and Cross-Reactivity**

To assess potential antibody cross-reactivity and shared exposure pathways, we used linear mixed-effects models to estimate associations between quantitative IgG and IgM responses to dengue virus (DENV), Zika virus (ZIKV), and chikungunya virus (CHIKV). Each antibody response was modeled as a continuous outcome, with responses to the other two viruses included as fixed-effect predictors. Household ID was included as a random intercept to account for clustering of individuals within households. The general model took the form:

Yᵢⱼ = β₀ + β₁X₁ᵢⱼ + β₂X₂ᵢⱼ + uⱼ + εᵢⱼ

where Yᵢⱼ is the antibody response of individual i in household j, X₁ and X₂ are the antibody responses to the other two viruses, β₀ is the intercept, β₁ and β₂ are fixed-effect coefficients, uⱼ represents household-level random effects (assumed to be normally distributed with mean 0 and variance σ²ᵤ), and εᵢⱼ is the residual error (assumed to follow a normal distribution with variance σ²). We also calculated intraclass correlation coefficients (ICC) for each model to estimate the proportion of total variance attributable to household-level clustering, using the formula:

ICC = σ²ᵤ / (σ²ᵤ + σ²)

These statistical analyses were conducted in R using the *lme4* and *lmerTest* packages for linear mixed-effects modeling.
