# Supplementary material for: High seroprevalence of antibodies to Dengue, Chikungunya, and Zika viruses in Dire Dawa, Ethiopia: A cross-sectional survey in 2024
Source: PLoS Negl Trop Dis. 2025 Jul 28;19(7):e0013357. doi: 10.1371/journal.pntd.0013357 (PMC12352872; doi:10.1371/journal.pntd.0013357)
Supplement: Attachment — Submitted filename: Additional Editor Comments_PLOSNTD.docx [file pntd.0013357.s003.docx]

Dear Editors and Reviewers;

Thank you for the helpful comments, critiques, and suggestions. We have now thoroughly revised the manuscript and have attempted to satisfactorily address each comment raised. We believe that the revised manuscript is much stronger as a result of these revisions. We also provide a point-by-point response below with our responses in ***bold blue italics***.

Reviewer #1: (No Response)

Reviewer #2: Would be beneficial to include the model equations and power calculation? Why were the number of individuals tested?

***We have included more text about the sample size now:***

***“We conducted a seroprevalence survey in Dire Dawa in March 2024. A total of 339 individuals were recruited for the survey. At the time of study planning, there were no reliable seroprevalence estimates for arboviruses in Dire Dawa. We therefore based our power analysis on an assumed 20% IgG seroprevalence, a 95% confidence level, and 5% absolute precision. Assuming a household-level clustered sampling design with an average household size of 4.5 individuals and an intra-class correlation coefficient (ICC) of 0.1, we estimated a design effect of 1.35. This yielded a target sample size of approximately 332 individuals. Although assuming 50% seroprevalence would have been more statistically conservative (as it maximizes required sample size), such an approach was not feasible given our resource constraints. We specifically targeted kebeles (administrative units) where local public health officials reported previous dengue outbreaks based on their clinical and field experience. While not derived from formal outbreak reports or geolocated case data, this strategy was intended to increase the likelihood of detecting a serological signal. Households were selected using a household registry and a table of random numbers. In each selected household, all individuals were invited to participate regardless of age.”***

***For the secondary, comparison survey in Addis Ababa – we did not use a formal power analysis. In this case we believed that true seroprevalence would be very near zero and therefore would require extremely large sample sizes in order to estimate a seroprevalence with any standard measure of accuracy. Instead, or goal was to test the same test kits in a place where we expected few to no test positives as a way of contextualizing the results we expected from Dire Dawa and in the absence of a lab confirmation of the tests. We’ve added the following text to the methods section in an attempt to better explain this now: “We recruited 180 individuals for this secondary survey, targeting two kebeles in Addis Ababa. The survey was not intended to generate precise prevalence estimates, but rather to serve as a pragmatic reference point by applying the same diagnostic tests in a setting with no known history of Aedes-borne arbovirus outbreaks. Given the anticipated low exposure, we expected few if any seropositive results. Our goal was to contextualize test performance in a lower-risk area given our absence of laboratory-based confirmatory diagnostics.”***

***We also provide more statistical details in the supplemental materials for sample size calculations and for the models used in this analysis.***

Why were only districts with historical dengue targeted. If it was for resources thats fine but justify.

***Thank you for this question. We have now added the following sentence to explain our logic in selecting kebeles where there was a known history of dengue outbreaks: “We specifically targeted kebeles (administrative units) where local public health officials reported previous dengue outbreaks based on their clinical and field experience. While not derived from formal outbreak reports or geolocated case data, this strategy was intended to increase the likelihood of detecting a serological signal. ”***

Should consider incorporating published sensitivity and specificity data for the serologic test into the data analysis to account for it.

***Thank you for this suggestion. We considered incorporating published estimates of sensitivity and specificity into our analyses but ultimately decided not to adjust our results for a few key reasons. First, validation data for the Chembio DPP® ZCD IgM/IgG test are limited, and most available figures come either from the manufacturer or from studies conducted in non-African settings. One relevant independent evaluation conducted in Thailand reported low sensitivity for IgG detection, particularly for dengue, which suggests that our estimates may in fact be underestimates of true seroprevalence. However, applying these figures to our dataset would likely lead to dengue IgG prevalence estimates exceeding 100%. Given these limitations, we have opted to report the raw results and clearly state the test’s limitations in the Discussion. We have added further text clarifying this issue and highlighting the need for future confirmatory studies (e.g., using PRNT) in East African populations to improve the accuracy of seroprevalence estimation.***

It does not make sense biologically to test for IgG predicting IgM (Line 238). Or justify it.

***The reviewer is correct. Our intention was not to imply causality but rather to assess statistical associations between all combinations of serological responses. The language used (“predictors” and “outcomes”) reflects the structure of the statistical models rather than any implied biological directionality. We’ve changed the wording from:***

***“IgG values were significant predictors of each IgM outcome, but their effect sizes were small.***

***to***

***“IgG values were statistically associated each IgM outcome, but their effect sizes were small.”***

***This change more accurately reflects our analytical goal of exploring correlations among serological markers. We interpret these associations as evidence that individuals with prior exposure to one virus may also have recent exposure to the same or a related virus, rather than any directional immune mechanism.***

Need a table showing how many people in each age were tested.

***Done. This is not included as Table 1.***

Reviewer #3: The objectives of the study are clearly stated. The population is clearly described and appropriate.

It is not completely clear what was the purpose of having a second serosurvey site. You present the general seroprevalence in Addis Ababa but did you use this information in any other way?

How was helpful? How was used?

***Thank you for this question. Our aim with the secondary serosurvey in Addis Ababa was to apply the same serological test kits in a setting where we expected little to no Aedes-borne arbovirus exposure, given the absence of documented outbreaks in that area. In the absence of laboratory-based confirmatory testing, this approach provided a pragmatic way to contextualize test performance and interpret results from Dire Dawa, where we anticipated—and subsequently observed—a higher burden of exposure. For instance, had we observed similarly high seroprevalence in both locations, it would have raised concerns about the interpretability of the test results. We have now added some text to the manuscript to help explain this logic: “ The survey was not intended to generate precise prevalence estimates, but rather to serve as a pragmatic reference point by applying the same diagnostic tests in a setting with no known history of Aedes-borne arbovirus outbreaks. Given the anticipated low exposure, we expected few if any seropositive results. Our goal was to contextualize test performance in a lower-risk area given our absence of laboratory-based confirmatory diagnostics.“***

No concerns relate to ethical or regulatory requirements.

**Results**

Reviewer #1: (No Response)

Reviewer #2: Need to present actual results of serological testing in table form not just estimates.

***Done. We have added these data as Table 1 in the main text now. The same data from Addis Ababa are also included in Supplemental Table 1.***

Reviewer #3: Yes, the results match the analysis plan. The results can be improved by presenting the crude numbers obtained for each serosurvey site and for clarifying is you are including just one site or both in each of your analysis and tables and graphs.

***Done. We have added these data as Table 1 in the main text now. The same data from Addis Ababa are also included in Supplemental Table 1.***

**Conclusions**

Reviewer #1: (No Response)

Reviewer #2: Need to better address/discuss the limitation of the sensitivity and specificity of the Chembio test to get at cross reactivity.

***We appreciate this important comment and have revised the manuscript to better acknowledge the limitations of the serological assays used. Although we cite previously published evaluations of the Chembio DPP Zika/Dengue/Chikungunya multiplex test (e.g., reference 25), we note that none of these validation studies were conducted in Africa. This is a key limitation, as test sensitivity and specificity may differ across regions due to differences in background immunity, circulating viruses, and co-exposure patterns.***

***Moreover, we did not have access to confirmatory laboratory assays such as PRNT or virus-specific ELISAs. As such, the true sensitivity and specificity of the assays in our study context remain unknown. We have updated both the Results and Discussion sections to reflect this uncertainty and have added further caution when interpreting seropositivity - particularly for Zika virus, where cross-reactivity with dengue virus is a well-documented concern.***

***These revisions aim to address the potential impact of cross-reactivity on our findings, while acknowledging that we cannot definitively distinguish between true ZIKV exposure and serological cross-reaction in this setting.***

Need to further elaborate on the mosquito biology and human behavior driving transmission in these areas to explain the results.
***Thank you for this suggestion. We agree that more detailed knowledge of mosquito biology and human behavior in this setting would greatly strengthen interpretation of the serological findings. However, one of the core motivations for this pilot study was the lack of such data in Ethiopia. While* Ae. aegypti *has been documented in the study area (reference 23, Yared et al 2024), few entomological or behavioral studies have examined the ecology or seasonality of arbovirus transmission in this region. As such, this study was designed as a foundational step—to generate preliminary serological evidence of exposure and build the rationale for future investigations that incorporate entomological surveillance and behavioral risk assessments.***

***To clarify this point, we have added a note to the discussion emphasizing that the absence of detailed mosquito ecology or transmission dynamics is a recognized gap and one that this study aims to help address.***

***“****Although Ae. aegypti has been documented in this region, data on vector abundance, seasonality, and human-vector contact patterns remain scarce. This pilot study was designed in part to address that gap and to generate preliminary evidence that can motivate more detailed entomological and behavioral investigations in future work. “*

Need to discuss the quantitative break-points/cut-offs for what a positive is. (Lines 214-218) Are these derived from PRNT assays, ELISA, how? Even though this is not the point of your study you are using the quantitative values in your analysis and thus need to introduce them.

***We have clarified in the revised text that the quantitative values used in this analysis are the raw numerical outputs from the diagnostic test reader provided with the commercial test kits. These values reflect the strength of the antibody signal detected by the assay but are not derived from PRNT or laboratory-based ELISA assays. While these values are used by the test reader to determine the binary classification of seropositivity, our cross-reactivity analysis specifically used the underlying quantitative values. This has now been explained explicitly in the revised text:***

***“We used the raw quantitative values generated by the test reader as the outcome variables in our cross-reactivity analyses, rather than relying solely on the binary classifications of seropositivity. These values reflect the magnitude of the antibody signal detected by the commercial test kit, though they are not derived from laboratory-based titration methods such as PRNT (plaque-reduction neutralization test) or ELISA (enzyme-linked immunosorbent assay). “***

Lines 240 - 247: need to better articulate/explain this in the conclusions. If you have household clustering of denv why not zikv? You would likely see it if there was enough ZIKV transmission or if sample size was larger.
***Thank you for this comment. We agree that the observed absence of household clustering in ZIKV responses warrants further explanation. We have now revised the relevant paragraph in the Results section to more clearly articulate potential interpretations of these findings. Specifically, we now note that the lack of detectable household clustering for ZIKV may reflect either a true absence of household-level transmission (consistent with a low force of infection), or a limitation in statistical power due to the relatively low number of ZIKV seropositive individuals in our sample. We also consider the possibility that some observed ZIKV IgG responses may be due to cross-reactivity with other flaviviruses, which could obscure spatial patterns tied to recent exposure.***

***“****The ICCs (intraclass correlation coefficients) indicated varying degrees of household clustering for the different viruses and immune responses. For DENV, we observed moderate clustering in IgG responses (ICC = 10.3%) and low clustering for IgM responses (ICC = 1.8%), suggesting some shared exposure or immunity patterns within households. CHIKV showed similar IgG clustering (ICC = 7.1%) and a stronger clustering effect in IgM responses (ICC = 14.0%). This may indicate recent transmission events that clustered in space and time. ZIKV responses showed no detectable household clustering for either IgG or IgM (ICC = 0 for both). This lack of clustering may reflect true absence of household-level transmission - consistent with a lower force of infection - or could be the result of low statistical power, given the low frequency of ZIKV seropositivity in our sample. If ZIKV transmission were ongoing, some degree of clustering might be expected; the lack of such clustering could suggest minimal circulation of the virus or that the observed IgG responses are driven by cross-reactive antibodies unrelated to recent, spatially structured exposure.* ***“***

Line 262: syntax- you are not "seropositive to CHIKV IgM" you are seropositive for IgM to CHIKV.
***Thank you for pointing this out. We’ve now corrected this throughout the manuscript.***

Line 269: citation 21 needs context/elaboration. This whole paragraph needs to be reworked to have direction. ***Thank you for this comment. Indeed, this paragraph included a typo and would benefit from more discussion. We have now edited the existing paragraph and added more text along these lines.***

Line 313: Seroprevelance does not indicate burden of disease when the viral infections are largly asymptomatic and do not produce disease. It would be better to discuss this as risk of infection. If you want to discuss potential burden without surveying symptomatic patients then incorporate published data on the rate of disease resulting from infection.

***We agree and have changed the wording to better reflect this distinction:
“While we cannot rule out the potential for cross-reactivity influencing our results, they are suggestive of a large risk of infection with Ae. Aegypti-transmitted viruses in a setting where this important vector of disease is known to be present. Repeated infections can in some cases lead to more severe disease outcomes, for example through ADE. More research into arboviral diseases and their mosquito vectors in this setting is warranted both to confirm our results and to develop a better understanding of the distribution of different arboviral infections, any potential age and gender associated risk factors, and all so that public health interventions can be formulated to address these diseases.”***

Reviewer #3: More discussion about how the known performance of the used test (especially specificity) helps supporting your results and your claim that Zika results are not an artifact of cross-reactivity with dengue.

***Thank you for this thoughtful comment. In the revised manuscript, we have clarified that our Zika seropositivity findings should be interpreted with caution. We acknowledge that cross-reactivity between flaviviruses, including dengue and Zika, is a known limitation in serological surveys. Without confirmatory diagnostics we cannot rule out the possibility that the Zika IgG results reflect cross-reactive responses rather than true past infection. Although we observed some patterns in the data that are consistent with Zika exposure, such as partial overlap in IgM responses and differences in signal intensity, these are not sufficient to draw firm conclusions. We now present these findings as hypothesis-generating and have avoided making strong claims about Zika circulation. The revised Discussion reflects this more cautious interpretation and emphasizes the need for future confirmatory work.***

**Editorial and Data Presentation Modifications?**

Reviewer #1: (No Response)

Reviewer #2: Need to present actual testing results by age and area not just estimates in table form. Put the results in tables.
***Done.***

Some minor syntax and grammatical errors.
***We have attempted to correct all syntax and grammatical errors.***

Stay consistent with metric units throughout.
***We have now dropped measurements in Fahrenheit and inches, leaving only metric measurements.***

A diagram explaining your cross reactivity hypothesis is needed.

***Thank you for the suggestion. In earlier drafts of the manuscript, we discussed possible cross-reactivity patterns in more detail. However, in response to reviewer feedback and to reflect the limitations of our data, we have now revised the manuscript to present these findings more cautiously. We emphasize that cross-reactivity between flaviviruses is well documented and acknowledge that our Zika findings may reflect this phenomenon. Rather than proposing a specific hypothesis or mechanism, we now describe the observed patterns as preliminary and hypothesis-generating. Given this more tentative framing, we believe that including a diagram could overstate the strength of our interpretation and imply greater certainty than is appropriate. We hope that future studies incorporating confirmatory assays will allow for more definitive exploration of these relationships.***

Reviewer #3: As Yellow Fever is also an arbovirus transmitted by Ae. aegypti of high interest in the African continent, and to make your paper easier to find for those interested in DENV, CHIKV and ZIKV, consider using the specific names of the viruses instead of “Aedes-borne arboviruses” in your title.
***We appreciate the reviewers point and have changed the title to: “High Seroprevalence of Antibodies to Dengue, Chikungunya, and Zika Viruses in Dire Dawa, Ethiopia: A Cross-Sectional Survey in 2024”***

Line 52: suggest including the word “virus” for each of them. “are” is repeated.
***Done.***

Line 55: you don’t need the word “viruses” here
***Corrected.***

Line 58: The current WHO clinical/severity classification is dengue, dengue without warning signs, and severe dengue. If you are including DHF and DSS because of country specific guidelines, I suggest you clarify that is the reason.

***Thank you for this observation. We were not using “severe” as a formal classification but rather as a qualitative indicator of disease outcomes (i.e. some infections have worse outcomes – including DHF and DSS). We have now clarified the language to distinguish between formal WHO classification and our more general reference to serious clinical outcomes. We acknowledge the current WHO classification of dengue and have revised the sentence accordingly to avoid confusion.***

***“DENV, endemic in tropical and subtropical regions, is responsible for an estimated 400 million infections annually, with a subset of cases progressing to dengue hemorrhagic fever and dengue shock syndrome (1).”***

Line 69: Zika infections
***Corrected.***

Line 73: Suggest IgM antibodies instead of ‘antibodies to IgM’
***Done.***

Line 75: Consider including specifics about the 4 dengue virus types here
***Thank you for this helpful suggestion. We have added a brief reference to the four dengue virus serotypes and clarified that infection with one serotype typically provides lifelong immunity only to that serotype. This addition helps contextualize the long-term persistence of IgG antibodies and introduces the potential for sequential infections with different serotypes, which we discuss in greater detail later in the manuscript in relation to disease severity and cross-reactivity.***

***“Dengue virus has four distinct serotypes (DENV-1 through DENV-4), and infection with one serotype typically provides lifelong immunity to that serotype but not to the others. “***

Line 78: Cross-reactivity is a concern for dengue and Zika viruses, but not for chikungunya
***We have updated this sentence to improve clarity:
Cross-reactivity, particularly between flaviviruses such as dengue and Zika, can complicate seroprevalence surveys that attempt to distinguish exposure to specific viruses (11).***

Line 80: consider adding additional details regarding the specific years for these outbreaks, this is specially important since you are doing age-related analyses
***We have added some text to address this now: “Several dengue fever outbreaks have been documented in Ethiopia since 2013, including reported outbreaks in Dire Dawa in 2013 and 2015, and in other parts of East Ethiopia since (12–15). Limited surveillance and diagnostic capacity mean that many cases likely go undetected or unconfirmed, making it difficult to precisely define the timing and extent of outbreaks. “***

Line 101: you include the total number of participants recruited in the results
***Thank you for the comment. Since this section serves to describe both the dataset and the methods used to generate it, we briefly reiterate the total number of participants recruited (updated total of 339) as part of the study population description. We believe this helps orient the reader before presenting the results and does not unnecessarily duplicate information from the methods.***

Line 104: It would be helpful to expand on this, did you have access to geolocation data of previous cases? City level official reports? Something else?
***We have updated the text with more description now: “We specifically targeted kebeles (administrative units) where local public health officials reported previous dengue outbreaks based on their clinical and field experience. While not derived from formal outbreak reports or geolocated case data, this strategy was intended to increase the likelihood of detecting a serological signal. “***

Line 106: Does “secondary” means anything specific here or refers to a second serosurvey? When was this survey conducted?
***Thank you for this helpful clarification prompt. We have revised the manuscript to clarify that “secondary” refers not simply to the second serosurvey chronologically, but to the fact that the Addis Ababa survey was not part of the primary study aim. Rather, it served as a pragmatic reference or control, designed to contextualize results from Dire Dawa by applying the same diagnostic tests in a lower-risk setting with no known Aedes-borne arbovirus outbreaks. We have also added the timing of the Addis Ababa survey (August 2024) to the text for clarity.***

Line 112: Do you have information on the approximated area and population you covered for each of the serosurveys? I am assuming you did not select the full area of either city as they are of a considerable size.
***Thank you for this important point. We confirm that we did not sample across the full extent of either city. Instead, we selected specific kebeles (administrative units) within each city based on local input and study feasibility. Unfortunately, we do not have precise population or area estimates for the specific kebeles included in the surveys. We have added a note in the manuscript to clarify this limitation.***

Line 114: I don’t think you are measuring exposure to Aedes aegypti with this study, I suggest modifying this sentence.

***Thank you for this important observation. We did not collect precise information on the geographic area or population size represented by the sampled kebeles in either Dire Dawa or Addis Ababa. As such, we have added a clarifying statement to the manuscript (Discussion section) noting this limitation. “We also do not have precise information on the total population size or geographic areas represented by the selected kebeles, which limits our ability to generalize these findings to the broader urban population.”***

Line 116: But how was this helpful or used for your analysis?
***Thank you for the question. The Addis Ababa survey served as a pragmatic reference point for interpreting seroprevalence in a lower-risk setting. Our aim was not to generate precise prevalence estimates but rather to contextualize findings from Dire Dawa, where prior dengue outbreaks had been reported. Addis Ababa differs markedly in climate, elevation, and ecological conditions and has no confirmed history of Aedes-borne virus outbreaks. The inclusion of this secondary site allowed us to assess the background level of seroreactivity. Importantly, had we observed similarly high seroprevalence in Addis Ababa, it would have raised concerns about test specificity or unrecognized widespread exposure. Instead, the low seropositivity we observed in Addis supports the interpretation that the higher levels in Dire Dawa reflect true transmission rather than assay artifact. We have clarified this rationale more explicitly in the revised manuscript.***

Line 119: Is this a fingertip prick test? Include this information in the methods.
***Yes, we used finger prick sampling for this study, and we have now clarified this in the revised methods section.***

Line 180: Suggest reporting your results in a consistent manner, either with % or with proportions throughout the document.
***Thank you for this observation. We have reviewed the manuscript to ensure that reporting is consistent within each context. For clarity, we use percentages when describing results in the narrative and use proportions for statistical model output and confidence intervals, which is common practice. We believe this dual format improves readability while maintaining precision in reporting model estimates. We have also checked the manuscript to ensure these formats are used consistently in their respective contexts.***

Line 202: Suggest reporting these results in increasing age groups (age 10, age 20, etc)
***Thank you for the suggestion. We have the option of presenting by virus or by age here. We would prefer to keep the organization by virus.***

Line 210: Is this for all participants in both serosurveys? This is not clear for the Dire Dawa results section starting in line 180.
***This is only for Dire Dawa, as the results from Addis Ababa are only for comparison and there were not sufficient results to do formal analyses with those data (from Addis). We hope that the clarifications on this that we have now added to the manuscript in other parts now make this more clear here as well.***

Line 124: this paragraph is confusing. Using negative and positive in a consistent manner (instead of “non positive”) might help with clarity.
***We have attempted to make this paragraph less confusing.***

Line 153: Chikungunya is an alphavirus and should not have any cross-reactivity concerns with the other two flaviviruses included in this analysis.
It is true that chikungunya is an alphavirus, and also that

***We agree that cross-reactivity between chikungunya virus (CHIKV), an alphavirus, and the flaviviruses dengue (DENV) and Zika (ZIKV) is unlikely. We have revised the manuscript to clarify this distinction and to explain that CHIKV was intentionally included in the modeling framework as a biologically distinct comparator. This approach allows us to contrast expected flavivirus cross-reactivity patterns with a virus for which no such cross-reactivity is anticipated. We now reference literature supporting the minimal likelihood of cross-reactivity between alphaviruses and flaviviruses, and we use CHIKV as a form of internal specificity check for the observed relationships among DENV and ZIKV.***

[***https://pubmed.ncbi.nlm.nih.gov/34703255/***](https://pubmed.ncbi.nlm.nih.gov/34703255/)

Line 217: Consider moving supplemental figure 2 to the main manuscript as this would help readers interpret your results in a much faster and easier manner.
***Thank you for this valuable suggestion. We have now moved Supplemental Figure 2 to Figure 1.***

Line 220: What does “reactive” means? Positive?
***Yes, it was meant to indicate seropositive. We have now reworded this section in an attempt to improve clarity.***

Line 257: Did you have infants under 6 months? What were their results for IgG? Thinking about maternal IgG transfer
***We do not have any participants under age 1 for this study, and did not record specific months of age.***

Line 269: Check this sentence, seems to be incomplete
***Yes, there was a typo here that has now been corrected. Thank you.***

Line 270: Not sure this information is relevant. Overall, this whole paragraph can be reorganized and reworded.
***We have now updated this entire section and hope that it is now more relevant and clear.***

Tables and Figures: Make sure that titles are complete, including place and time.
***Done.***

Figure 1: Since you are reporting the probability by age using 10, 20, 30, etc, consider having those ages as your x axis markers or adding something in the lines that guides to reader to the predicted probability of those ages.

***Done. We have now edited these figures in an attempt to make them more easily interpretable and aligned with the text.***

**Summary and General Comments**

Reviewer #1: Major commetns:
The introduction does not adequately contextualize arboviral epidemiology in Ethiopia, specifically for Zika virus. More detail on existing studies or surveillance gaps is needed.

***Thank you for this comment. We have now attempted to address these concerns in the introduction and discussion sections of the revised manuscript. In general, very little has been done with regard to arboviral epidemiology in Ethiopia, even more so with the normally asymptomatic Zika, and we hope that the current work can be a step towards improving this gap. We reference a recent genomic epidemiological paper (Abera et al 2025) that is insightful along these lines and have now included it as a reference as well.***

While the manuscript explains the selection of Dire Dawa due to its history of arbovirus outbreaks, it does not sufficiently justify the choice of Addis Ababa as the comparison site. Why was Addis Ababa selected despite its lack of reported outbreaks? Provide a clearer rationale for its inclusion and discuss how this might affect the study's conclusions.

***Thank you for this comment and please see our responses above to the same question. Briefly, we included Addis Ababa as a type of control – especially given our lack of a laboratory confirmed control.***

The manuscript briefly discusses household-level clustering effects but does not explore the implications of this finding. For example, could household clustering suggest shared environmental risk factors, such as mosquito breeding sites or socioeconomic status? Discuss how these findings could guide targeted interventions.
***Yes, thank you for this comment and suggestion. We have now added the following paragraph to the discussion section: “We also found evidence of household level clustering for DENV and CHIKV IgG responses and for CHIKV IgM responses. In contrast, ZIKV responses showed no measurable household clustering. These patterns could reflect differing transmission dynamics or the timing of exposure across viruses. Household clustering in IgG or IgM responses may point to shared environmental exposures such as mosquito breeding sites near houses, or to household-level behaviors and socioeconomic factors that influence risk of infection. Future interventions or surveillance efforts might benefit from recognizing and targeting such within-household transmission patterns.”***

Although the study acknowledges potential cross-reactivity between DENV and ZIKV IgG results, it does not explore how this might affect public health recommendations. Could these findings lead to diagnostic challenges or misclassification in clinical and epidemiological settings? Consider elaborating on strategies to address such issues.

***We appreciate this important suggestion. In the revised manuscript, we have now expanded our discussion of the implications of serological cross-reactivity for surveillance and diagnosis. Specifically, we note that cross-reactive antibody responses between DENV and ZIKV may result in misclassification of past exposures, especially in the absence of molecular confirmation or PRNT-based assays. This can affect outbreak detection, risk mapping, and eligibility for dengue vaccination. We also highlight the need for local validation of diagnostic assays and careful interpretation of serological data in settings where multiple flaviviruses may circulate. These additions are found in the Discussion section, where we now split the prior paragraph on cross-reactivity to include a new paragraph:***

***These complexities have direct implications for surveillance and clinical interpretation. In the absence of molecular confirmation or PRNT-based serotyping, distinguishing true infections from cross-reactive responses is difficult. Misclassification could compromise seroprevalence estimates and introduce uncertainty into outbreak detection, risk mapping, and vaccine eligibility assessments. This highlights the importance of continued assay validation, ideally in local populations, and careful interpretation of serological survey data in flavivirus-endemic settings.***

The age-specific seroprevalence results are intriguing, particularly the rapid increase in IgG seropositivity for DENV by age 10. However, the manuscript does not delve into why children might exhibit such high seroprevalence early in life. Discuss potential exposure routes, such as maternal antibody transfer, vector density, or environmental factors.

***Thank you for your helpful comment regarding the age-specific seroprevalence patterns. We have now added a paragraph to the Discussion section that addresses possible explanations for the high IgG seropositivity for dengue among young children. Specifically, we discuss how these patterns are consistent with recent or ongoing transmission and how comparisons with CHIKV and ZIKV age-patterns may reflect differences in force of infection or timing of virus introduction. We also note that the smooth age-seroprevalence curves for dengue are consistent with endemic circulation rather than sporadic outbreaks. Finally, we include a caution regarding interpretation of ZIKV results due to potential cross-reactivity with DENV. This new paragraph has been added immediately following the discussion of sampling design and generalizability in the Discussion section.***

***“The observed age-specific IgG seroprevalence patterns offer important epidemiological insight. The rapid rise in DENV IgG positivity by age 10 likely reflects active or recent transmission, as seropositivity among young children must result from infections within their relatively short lifespans. In contrast, slower age-related increases in CHIKV or ZIKV IgG suggest a lower force of infection or more recent introduction of these viruses. The smooth, progressive increase in DENV IgG with age also supports the interpretation that exposure is accumulating gradually, consistent with endemic transmission rather than isolated outbreaks. While maternally derived antibodies may explain some reactivity in very young children, the broader pattern suggests widespread exposure during early childhood. Although the ZIKV findings are also intriguing, we remain cautious in interpreting those results due to the potential for cross-reactive antibody responses with DENV.”***

The manuscript refers to seroprevalence studies from other regions but does not perform a detailed comparison. How do the findings from Dire Dawa and Addis Ababa align or differ from seroprevalence rates reported in other African countries or similar ecological settings?

***Thank you for this important observation. We have now expanded the Discussion section to include a more detailed comparison of our findings with prior seroprevalence studies in Ethiopia and across Africa. Specifically, we highlight how most previous studies relied on febrile or hospital-based populations, which may not reflect broader community-level exposure. In contrast, our study used a population-based sampling strategy in an area with known outbreaks, allowing us to estimate seroprevalence in a more representative sample. We compare our findings to results from both a large nationally representative PRNT-based survey and a community-level survey in Gambella, discussing the potential influence of cross-reactivity and lack of confirmatory testing. We also emphasize that the secondary survey in Addis Ababa was intended solely as a pragmatic control and not a primary comparison site. These additions are intended to clarify the contextual significance of our results and the ways in which they both align with and differ from other findings across the region.***

Minor comments:
Many mistakes in the manuscript. Here is a part of it.
Line 31: “that that”
Line 52: “are are”
Line 55: “DENV, CHIKV, and ZIKV viruses”
Line 68: “Dengue and chikungunya infections”
Line 242: “DENGV”
Line 244: “Zika”
Line 255: “DENGV”
Line 362: lack “Figure 2”]

***We have gone through and corrected these errors/typos and have attempted to correct all throughout the manuscript.***

Reviewer #2: Study has potential for meaningful publication highlighting the need for improved arboviral surveillance on the African continent but is missing critical discussion on the limitations of the serological test used in the study, data presentation, and articulation of what biologically is driving the results. Also, justification of various methodologies is needed.

***Thank you for your helpful comments and suggestions. We have now attempted to improve the discussion, including around the limitations of the serological test being used and what is driving the results. We also attempt to better explain the logic behind our methodological approaches.***

Reviewer #3: This manuscript by Parker et al describe the results of dengue, chikungunya and Zika seroprevalence surveys conducted in Ethiopia. The information included in the manuscript is of interest for the field as very limite information on these arboviral diseases transmission is available for the region and the country. Overall, the manuscript is well written, althought it can benefit from some clarifications in the methods and results sections, and additional discussion details. I have included some specific comments for the authors.

***Thank you for your helpful comments and suggestions. We’ve now extensively revised the manuscript, including improving and clarifying details about the methods, results, and increasing the discussion section.***
